# Performance of a novel risk model for deep sternal wound infection after coronary artery bypass grafting

**DOI:** 10.1038/s41598-022-19473-1

**Published:** 2022-09-07

**Authors:** Bianca Maria Maglia Orlandi, Omar Asdrúbal Vilca Mejia, Jennifer Loría Sorio, Pedro de Barros e Silva, Marco Antonio Praça Oliveira, Marcelo Arruda Nakazone, Marcos Gradim Tiveron, Valquíria Pelliser Campagnucci, Luiz Augusto Ferreira Lisboa, Jorge Zubelli, Sharon-Lise Normand, Fabio Biscegli Jatene

**Affiliations:** 1 Department of Cardiovascular Surgery, Instituto do Coração do Hospital das Clínicas da Faculdade de Medicina da Universidade de São Paulo (INCOR), São Paulo, São Paulo Brazil; 2 Department of Health Care Policy, Harvard Medical School, Boston, USA; 3 Department of Cardiovascular Surgery, Hospital Samaritano Paulista, São Paulo, São Paulo Brazil; 4 Universidad de Costa Rica, San José, Costa Rica; 5 Instituto de Matemática Pura e Aplicada (IMPA), Rio de Janeiro, Brazil; 6 Department of Cardiovascular Surgery, Beneficência Portuguesa de São Paulo, São Paulo, São Paulo Brazil; 7 Faculdade de Medicina de São José do Rio Preto, São José de Rio Preto, São Paulo Brazil; 8 Department of Cardiovascular Surgery, Irmandade da Santa Casa de Misericórdia de Marília, Marília, São Paulo Brazil; 9 Department of Cardiovascular Surgery, Irmandade da Santa Casa de Misericórdia de São Paulo, São Paulo, São Paulo Brazil; 10 Khalifa University, Abu Dhabi, United Arab Emirates; 11 Department of Biostatistics, Harvard TH Chan School of Public Health, Boston, USA

**Keywords:** Risk factors, Outcomes research, Preventive medicine

## Abstract

Clinical prediction models for deep sternal wound infections (DSWI) after coronary artery bypass graft (CABG) surgery exist, although they have a poor impact in external validation studies. We developed and validated a new predictive model for 30-day DSWI after CABG (REPINF) and compared it with the Society of Thoracic Surgeons model (STS). The REPINF model was created through a multicenter cohort of adults undergoing CABG surgery (REPLICCAR II Study) database, using least absolute shrinkage and selection operator (LASSO) logistic regression, internally and externally validated comparing discrimination, calibration in-the-large (CL), net reclassification improvement (NRI) and integrated discrimination improvement (IDI), trained between the new model and the STS PredDeep, a validated model for DSWI after cardiac surgery. In the validation data, c-index = 0.83 (95% CI 0.72–0.95). Compared to the STS PredDeep, predictions improved by 6.5% (IDI). However, both STS and REPINF had limited calibration. Different populations require independent scoring systems to achieve the best predictive effect. The external validation of REPINF across multiple centers is an important quality improvement tool to generalize the model and to guide healthcare professionals in the prevention of DSWI after CABG surgery.

## Introduction

Although outcomes of cardiovascular surgery have improved over time, the incidence of deep sternal wound infection (DSWI) remains an important issue. Recently published data showed an incidence of DSWI ranging between 1.3 and 1.6% in patients undergoing CABG alone^1,2^. Morbidities associated with DSWI include prolonged hospital stays, increased use of antibiotics and consequent increased costs. Patients with DSWI had a threefold increase in hospitalization costs compared with patients without DSWI. In addition, mortality from this complication continues to concern.

Prediction models for DSWI exist but may not be generalizable in different geographic settings. Incidence of DSWI in developed countries may be lower, considering their resources, application of best practices to avoid these complications and population characteristics. Moreover, models that include more than one cardiac surgery type may have heterogeneous in case-mix, thereby limiting the discriminant ability^3–7^. Healthcare systems, patients’ characteristics, and quality protocol adherence varies widely between institutions.

The Magendanz score, a specific prediction tool for Mediastinitis, was based on data from a single center for which some risk factors were missing, data quality assessment was incomplete, and no external validation in independent samples was conducted, thus impacting generalizability^4,5^. Methodologically, addressing the observational nature of the data due to lack of randomization of patients to institutions and missing information for key patient-level variables are two critical challenges requiring attention^6^.

Valid statistical approaches to missing confounder information include multiply imputing the missing information, inverse-probability weighting, or comprehensive sensitivity analyses. The inclusion of more confounders and multiple imputation of missing information should enhance the predictive performance of a model^3,7,8^. We hypothesized that specific clinical characteristics, including pre and intraoperative factors, would be associated with better accuracy to predict DSWI on a multicentric registry. This study aimed to develop and validate a prediction model, the REPINF, using data from the Cardiovascular Surgery Registry of the state of São Paulo, Brazil (REPLICCAR II), and compare with the validated model STS.

## Methods

The REPLICCAR II study is an observational, multicenter, prospective cohort study (9 hospitals in the state of São Paulo) conducted between August 2017 and June 2019. The Ethical Committee Board of the Heart Institute of the Hospital das Clínicas, Faculty of Medicine, University of São Paulo, Brazil approved this study as a sub-analysis of the REPLICCAR project (CAPPesq: 2.507.078). Thus, informed consent was waived due to the analysis of pre-established data logs.

We declare that all methods were performed in accordance with relevant guidelines and regulations. All consecutive patients over 18 years undergoing isolated CABG surgery (first cardiac surgery) constituted the sample. The indications for CABG surgery were according to guidelines^9^. All patients received antibiotic prophylaxis at least one hour before skin incision according to institutional policies.

The variables included in REPLICCAR II were defined using the STS ACSD (Adult Cardiac Surgery Database) collection tool (version 2.9, 2017). Approximately 760 variables were collected preoperatively, intraoperatively, and postoperatively, and included risk factors, clinical and laboratory characteristics, and complications of surgery. The data were collected using a secure web application for building and managing online surveys and databases, the REDCap platform (Research Electronic Data Capture, https://www.project-redcap.org/).

The participating hospitals, their researchers, and data managers participated in meetings and data training before and during the data collection period. Data were audited twice by the REPLICCAR II team to evaluate the accuracy and validity of the information collected by the trained data managers^10^.

A trained surgical clinical nurse reviewed the infection criteria and definitions following the infection control surveillance system (Standard CDC National Healthcare Safety Network definitions following the National Healthcare Safety Network—NHSN)^11^. All infections involving the subcutaneous tissue to the mediastinum within 30 days following CABG surgery were considered DSWI. This involves fascia and muscle layers as well as organs, spaces and/or deep soft tissues. “Mediastinitis” refers to an infection of the mediastinum, which can be caused by different etiologies, including DSWI following sternotomy^11^. A computed tomography imaging study was performed in all patients with suspected DSWI and/or Mediastinitis for diagnostic confirmation. The infection control services of the participating hospitals on REPLICCAR II perform routinely the active surveillance to report surgical wound infections that progressed to deep planes and Mediastinitis. Data were verified by a specialist nurse from the coordinating center while carrying out her doctoral project. Thus, only cases diagnosed with the definitions and criteria of the CDC–NHSN were included in our analysis. The reference was reviewed and changed for the one used for the infection control services during the study. Patients who have a fascia or muscle affected by an infection during hospitalization often receive surgical wound debridement, antibiotics and negative-pressure wound therapy (vacuum-assisted closure) to prevent mediastinitis.

### Approach

#### Confounders and predictors

First, we eliminated all variables missing in more than 30% of the patients because the imputed values would be driven by the imputation model. We next identified variables related to incidence of DSWI in the scientific literature and found 160 variables in our database. Of these, 55 variables with statistical association or clinical significance for DSWI were considered as predictors (supplementary Table 1). Therefore, the variables were chosen for the initial analysis according to their relationship with the scientific literature and subsequently for their statistical significance, all of this given the multifactorial nature related to infections. Our goal was to build a model that was the most rational and at the same time scientifically robust, including both pre and intraoperative variables.

#### Treating missing data with multivariate imputation

We used chained equations (MICE) to impute missing data and created 10 imputed datasets.

Sample distribution was captured with histograms and descriptive statistics.

#### Statistical analysis

The training sample was created using the REPLICCAR II database that included 4,085 patients. Information from an additional 498 patients from a different set of hospitals was assembled to create an external validation set (from 2015 to 2016). The model development for variables selection and regularization was performed with the least absolute shrinkage and selection operator (LASSO) logistic regression tenfold cross-validation, to enhance the prediction accuracy and interpretability of the statistical model produced.

We calculated the area under the receiver operating characteristic curve (*c-index*) to evaluate the discriminatory performance of the model and calibration in-large (CL) containing the observed and predicted values (ratio of observed/predicted). The discriminative ability was also evaluated by net reclassification improvement (NRI) and integrated discrimination improvement (IDI)^12^. The results were plotted to compare the new model (REPINF) with the STS in both the training and validation databases.

We follow the guidelines recommended in the Transparent Reporting of a multivariable prediction model for Individual Prognosis or Diagnosis statement checklist (TRIPOD)^13^.

### Ethics approval

This study was submitted and approved by the Ethics Commission for Analysis of Research Projects (CAPPesq) under number 2016/15163-0. The free and informed consent was dismissed due to the analysis dealing with pre-established data logs.

## Results

Nine hospitals started data collection, but 7 hospitals actively participated during the 2 years of the project. After exclusion of the patients from 2 hospitals (n = 53), our final sample size was 4085 patients undergoing isolated CABG surgery as the first cardiac surgery. The mean age was 63.3 years (95%CI 62.9–63.5) and 74% were male. The mean body mass index (BMI) was 27 kg/m^2^ (95%CI 26.9–27.2) and common comorbidities included diabetes (49%), hypertension (88%), dyslipidemia (62%) and previous myocardial infarction (52%). The baseline characteristics and missing percentages are described in supplementary Table 2. After excluding variables with more than 30% missing, 5% of all patients had at least one missing variable in the REPLICCAR II database (n = 4085 and 160 variables).

The incidence of DSWI during 30 days from surgery was 2.47% (n = 101). We observed 104 deaths, a competing risk for DSWI, within 30 days (3.1%); of these, 3 patients died with DSWI in the period (2.9%). Characteristics between infected and non-infected patients are described in Table 1.

**Table 1:** Baseline characteristics of patients undergoing isolated CABG surgery with and without DSWI (n = 4085). REPLICCAR II, São Paulo, Brazil, 2017–2019.

	DSWI			
	Yes (n = 101)		No (n = 3984)	
	n	%	n	%
Age (years)^a^	63.6 ± 9.5		63.2 ± 9.2	
Gender male	50	49.5	2984	74.9
BMI (kg/m^2^)^a^	29 ± 5.5		27.4 ± 4.3	
Diabetes	72	71.3	1938	48.6
Hemoglobin (mg/dL)^a^	12.6 ± 1.86		13.5 ± 1.79	
Hematocrit (%)^a^	38 ± 5.1		40 ± 4.9	
NYHA ≥ III	3	3.0	140	3.5
Three-vessel disease	15	14.9	1060	26.6
**Surgery status**				
Elective	54	53.5	2600	65.3
Urgency	43	42.6	1371	34.4
Emergency	4	4.0	13	0.3
Lowest intraoperative temperature (°C)^a^	33.2 ± 1.9		33.8 ± 1.9	
Surgery duration (hours)^a^	5.9 ± 1.7		4.7 ± 1.6	
CPB time (minutes)^a^	87.1 ± 31.6		75.5 ± 29.2	
Anoxia time (minutes)^a^	69.8 ± 30.8		58.4 ± 24.8	
BITA	19	18.8	450	11.3
Intraoperative high glucose (mg/dL)^a^	205.1 ± 61.4		179.6 ± 59.9	
Intraoperative blood transfusion	30	29.7	700	17.6

BMI: body mass index; MI: myocardial infarction; NYHA: New York Heart Association; CPB: cardiopulmonary bypass; BITA: bilateral internal thoracic artery.

^a^Mean ± SD.

### Model development

**Figure 1:**
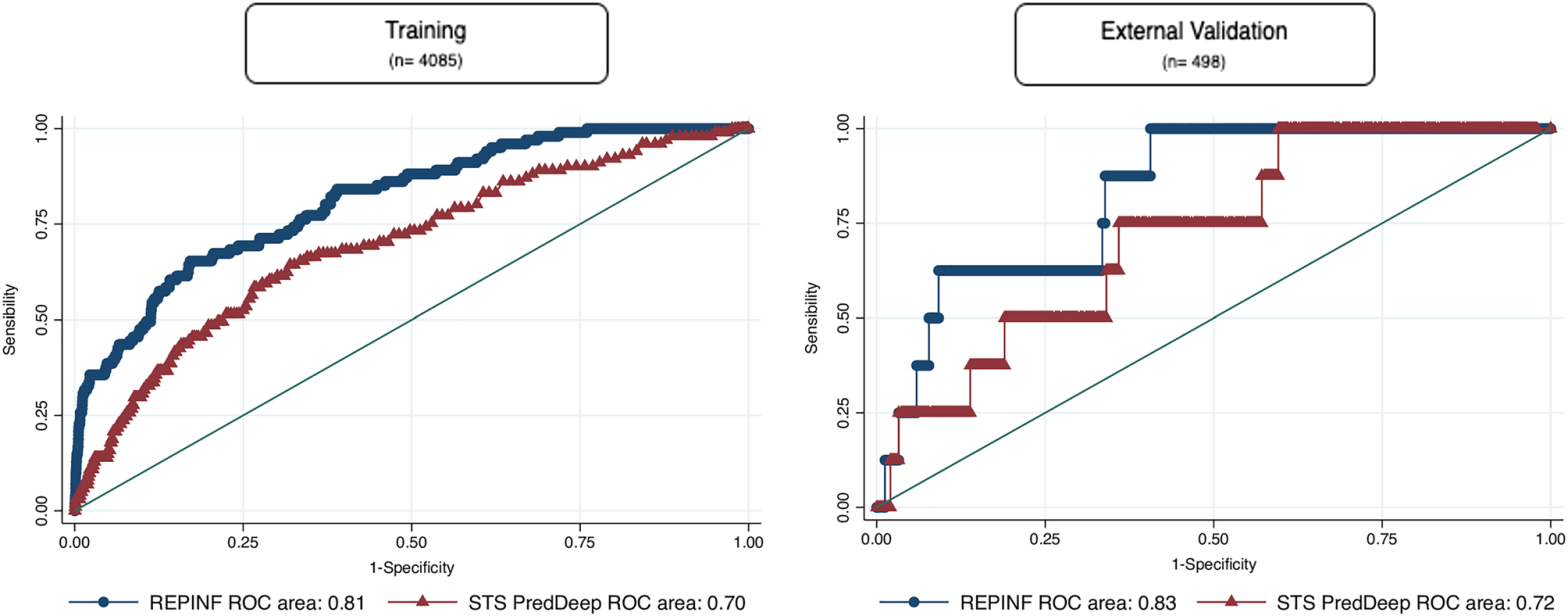
Receiver operating characteristic curve (ROC; c-index) in the external validation sample of the REPINF and STS in patients undergoing isolated CABG. Sao Paulo, Brazil, 2017–2019.

**Figure 2:**
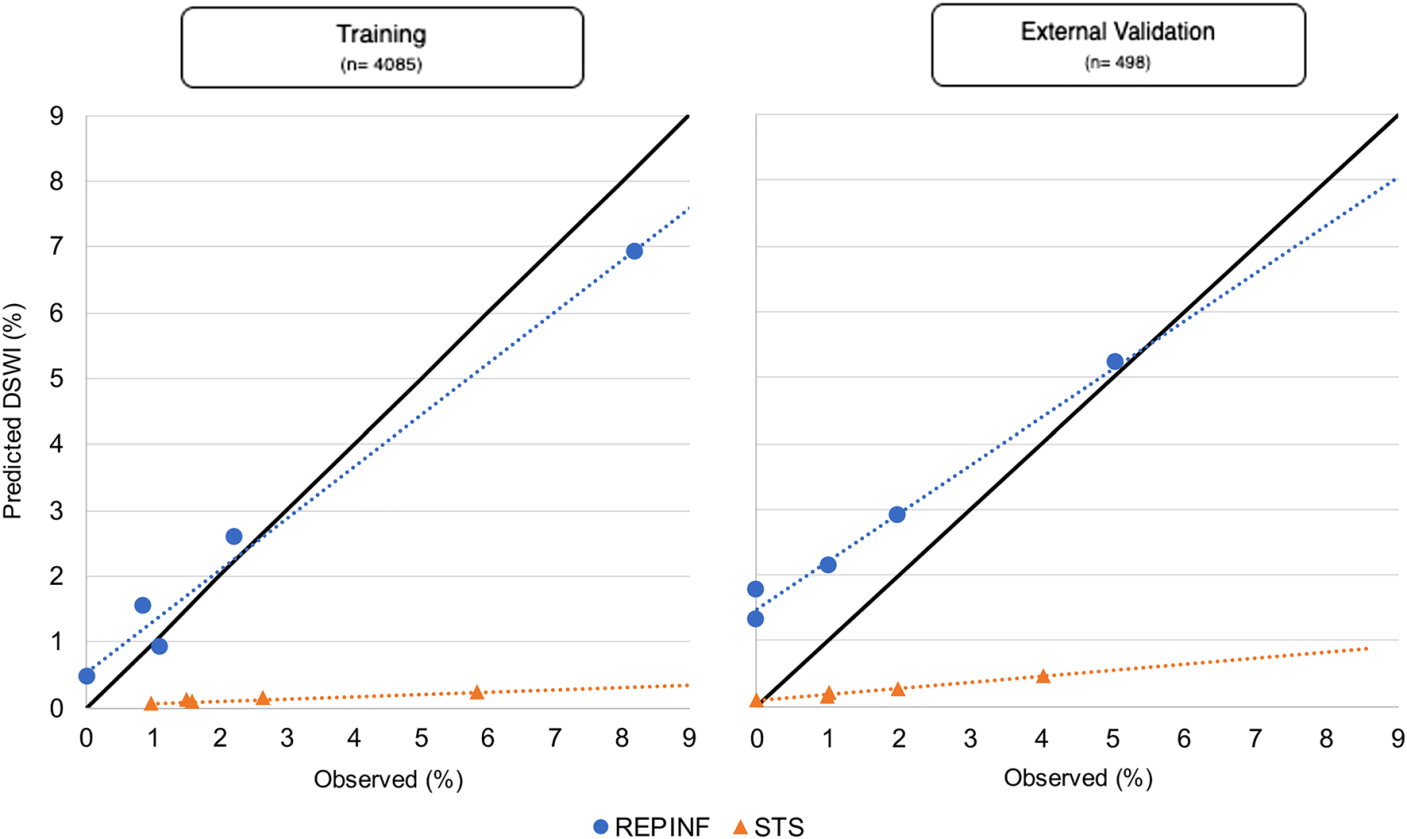
Calibration in-the-large plot on training and external validation. REPINF, Sao Paulo, Brazil, 2017–2019.

Out of a total 55 of variables related to pre- and intraoperative factors, 7 were included in the Lasso modeling after tenfold cross-validation (Table 2). In the training sample (n = 4085), the REPINF had a *c-index* of 0.81 (95%CI 0.77–0.86) compared to STS *c-index* of 0.70 (95%CI 0.64–0.75) (Fig. 1). The predicted mean for DSWI was 0.12% (SD = 0.08) using the STS. The calibration in-the-large plot (Fig. 2) demonstrated that STS predictions tended to underestimate the DSWI risk in our sample.

**Table 2:** LASSO logistic regression tenfold cross-validation coefficients. REPINF, REPLICCAR II, São Paulo, Brazil, 2017–2019.

Covariates	Coefficients	Logistic regression standard error
Female gender	0.246	0.267
Body mass index	0.041	0.025
Diabetes	0.134	0.279
Hemoglobin	− 0.182	0.236
Surgery emergency status	0.132	0.793
Surgery duration	0.433	0.091
Bilateral internal thoracic artery used	0.020	0.308
Constant	− 3851	–

### External model validation

The validation database included 498 patients undergoing isolated CABG during 2015–2016. The mean age was 61.7 (SD = 9.5), 78.5% were male, 54.6% had diabetes and 88% had hypertension. Relative to the STS, REPINF demonstrated improved classification with a NRI of 29% (Table 3) and, the discrimination IDI was 0.065 (6,5%). The incidence of DSWI in this sample was 1.61%. The STS model predicted 0.24% (SD = 0.15) of DSWI events and, REPINF 2.65% (SD = 1.58). The *c-index* was 0.83 (95%CI 0.72–0.95) and 0.72 (95%CI 0.56–0.88) for REPINF and STS in the external validation sample, respectively.

**Table 3:** Reclassification data table with quintiles for net reclassification improvement (NRI). REPINF, REPLICCAR II, São Paulo, Brazil, 2017–2019.

Event		REPINF						
		Quintile	1	2	3	4	5	Total
No	STS	1	379	217	138	67	36	837
		2	223	221	175	137	63	819
		3	128	181	172	184	123	788
		4	71	132	203	187	186	779
		5	16	57	122	224	342	761
			817	808	810	799	750	3984
Yes	STS	1	0	2	3	1	2	8
		2	0	3	0	4	6	13
		3	0	2	2	2	6	12
		4	0	1	0	4	16	21
		5	0	1	2	7	37	47
	Total		0	9	7	18	67	101

It is important to note that EuroSCORE II and STS are the most used worldwide to predict mortality risk after cardiac surgery. However, only the STS model has a validated index to predict the risk of DSWI. Therefore, our research team decided to compare our model only with the STS in order not to bring the comparison bias if we choose to use the EuroSCORE II since our outcome would not be mortality but DSWI. Below, we show in Table 4 some basic characteristics between the STS, REPINF and EuroSCORE II models.

**Table 4:** Comparison of baseline characteristics of STS, REPINF and EuroSCORE II models, São Paulo, Brazil, 2017–2019.

Score	STS	REPINF	EuroSCORE II
Timeframe for data collection	Jan 2002–Dez 2006	Aug 2017–Jul 2019	12 weeks (May–Jun 2010)
Study population	774,881 isolated CABG procedures	4085 isolated CABG procedures	22,381 major cardiac procedures
Multicentric	Yes, 819 participating centers	Yes, 7 participating centers	Yes, 154 participating centers
Statistical approach	Regression modeling	LASSO regression	Multivariate Logistic Regression
Outcome	Mortality (operative and in-hospital)	DSWI (30 days after surgery)	Mortality at the base hospital
Secondary outcomes	Renal failure, stroke, reoperation for any cause, prolonged ventilation, deep sternal wound infection, composite major morbidity or mortality, prolonged length of stay (> 14 days), and short length of stay (< 6 days and alive)	No	Mortality at 30 and 90 days
Discrimination DSWI (c- index)	Yes, STS PredDeep (c-index training: 0.71 and validation: 0.69)	Yes, REPINF (c-index training: 0.81 and validation: 0.83)	No

## Discussion

The development of prognostic models that combine patient characteristics, risk profiles, and surgical practice to produce predictions about future outcomes allow informed clinical decision-making^8^. Risk models can be used for quality measurement, clinical practice improvement, voluntary public reporting, and research.

The STS systematically underestimates the risk of infection in CABG patients, possibly due to the low rate of this complication for this type of procedure (less than 0.5%), yielding a c-index of 0.68 for CABG surgery patients^3^. The REPINF demonstrated better discrimination (IDI = 6,5%) and net reclassification improvement (NRI = 29%) compared to STS model.

Differences in health management and performance across countries may also affect model discrimination. The MagedanzSCORE^4^ (2010) was created using information from a single Brazilian institution among adults (n = 2809) undergoing isolated CABG and valve surgery. The score was developed and validated in the same population and thus provides overly optimistic model performance metrics^5^.

In this study, the incidence of DSWI was 2.47% (30-day follow-up) and the prognostic model was restricted to patients undergoing primary isolated CABG. Of 55 variables candidates in REPINF, 7 emerged from the LASSO logistic regression: female gender^14–16^, BMI^14,17–19^, diabetes^1,17–19^, hemoglobin^18^, emergency surgery status^18,20,21^, surgery duration^18,21^ and bilateral internal thoracic artery (BITA) used^22–24^. All variables included in the LASSO regression in the new model have already been described as risk factors for DSWI. In fact, the REPINF model included intraoperative variables already described for DSWI endpoint, as surgical timing^18,21^ and use of BITA^22–24^.

A recent prospective multicentric study with 16 centers of cardiac surgery in 6 European countries (England, Finland, France, Germany, Italy and Sweden) reported an incidence of DSWI of 2.5% and the following independent predictors: female gender, BMI ≥ 30 kg/m^2^, estimated glomerular filtration rate < 45 mL/min/1.73 m^2^, diabetes, chronic lung disease, preoperative atrial fibrillation, critical preoperative state and BITA grafting. The model achieved better discrimination than the usual scores (Alfred Hospital Risk Index, Friedman Score, and Brompton-Harefield Infection Score). Compared to these values, improvements in discrimination (IDI) ranged from 1.2 to 2.1%, but were not compared with the validated cardiac surgery model STS^24^.

A single ‘‘calibrated model’’ to make predictions across patients undergoing many different surgery types is challenging^25,26^. Our model achieved better accuracy than the STS having *c-indices* of 0.83 (REPINF) and 0.72 for STS in the validation cohort. It is important to note that the external validation database corresponds to an active participant in STS reports since 2014, which is center that is likely not representative of all Brazilian hospitals. The STS systematically underestimated DSWI in both the internal and external validation datasets (Calibration: Fig. 2). Our score overestimated risk in external validation cohort for those at the lower end of the risk scale. This overestimation may be related to the largest volume of patients coming from the public health system used for the elaboration of the REPINF. For validation, the REPINF was evaluated in a specific population of private network patients. This may have influenced REPINF to overestimate the risk of infection in the validation sample.

Calibration is an important aspect in models constructed for predictive purposes. It is necessary to keep data collection guided by rigorous quality registries and criteria to achieve and maintain the best accuracy in predictions, considering that this information may be often contaminated by noise^26^. To improve calibration, risk scores should be adjusted for the case-mix of hospitals, with recalibration or remodeling being recommended^27^. Adding more variables and optimizing estimates of improvement may increase model performance but at the same time cause overfitting. REPINF model was created considering these situations, where all variables included for LASSO regression were associated with DSWI creating a difficult task for the variable selection.

LASSO was originally formulated for linear regression, and it’s applied in statistics and machine learning for variable selection and regularization. Before LASSO, the stepwise approach is the most widespread method for choosing covariates. Also, LASSO improves prediction error by shrinking the sum of the squares of the regression coefficients to be less than a fixed value to reduce overfitting^26,28^.

Accurate information is essential to access patient’s prognosis, which simultaneously considers a number of factors and provides an estimate of the patient’s absolute risk of an event and, for DSWI, it is a great challenge. Clinicians and surgeons need an accurate risk prediction for decision support, quality of care assessment, and patient education. Continuous evaluation of the model performance is important to ascertain that the classification performance does not degrade with time. Some models are redeveloped periodically to adjust for temporal trends. Recently, STS updated the model to predict mortality in children following cardiac surgery using the proposed machine learning method^29–33^.

We suggest that REPINF score should be estimated when the patient arrives to the intensive care unit. This moment becomes fundamental in the management of patients after CABG. By this way, the professional team would be able to establish a clear plan of care based on the patient risk, minimizing thereby the potential complications, and reducing costs and hospital length of stay. Also, specific protocol may be developed by the infection control team^34^. More investigation should be performed to determine cut-offs on risk classification and timing for the application such preventing strategies. This paper describes the development and validation of the deep sternal wound infection model for CABG patients. According to the medical literature, factors related to intraoperative timing are also associated with Mediastinitis, so we included these variables in our registry for analysis. Limitations related to data completeness and accuracy were carefully addressed during quality audits for all institutions. Still, some important clinical aspects were not evaluated and could increase the sensibility and precision of the model, for example, the use of pedicled or skeletonized harvesting conduits, glycated hemoglobin, albumin, bilirubin, and variables related to DSWI treatment (fluid or tissue culture, antibiotics, wound intervention, bandages, and others). In our institutions, the infection control service follows these data for epidemiological surveillance, and to guide preventive protocols according to the CDC surgical site prevention manuals^34^. Future studies should consider all possible detailed information and recommend standard prevention interventions to avoid bias and increase accuracy.

Another important issue is related to the DSWI detection method (30-day follow-up), which may vary across institutions^24,25^. In our study, this limitation was controlled by having trained researchers to make contact 30 days after surgery with each patient, with only 5.97% of incomplete follow-up.

In summary, this study considered a structured, standardized approach to model development, and validation to identify factors to help multidisciplinary teams prevent DSWI after CABG. More studies should be performed to validate these findings, but we suggest that REPINF, as well as the STS prediction models^35^, provides the highest generalizability for future data. Thus, it’s proven that different populations require independent scoring systems to achieve the best predictive effect.

## Supplementary Information

Supplementary Tables.

## Data Availability

The data generated during the current study are not publicly available due to ethical restrictions; patients did not consent to their deidentified data being publicly shared but are available on reasonable request to the Scientific Committee Director Renata do Val (renata.doval@incor.usp.br; https://www.incor.usp.br/sites/incor2013/index.php/16-pesquisa/comissao-cientifica/158-fale-conosco).
