# Author Correction: Performance of a novel risk model for deep sternal wound infection after coronary artery bypass grafting

**DOI:** 10.1038/s41598-022-23963-7

**Published:** 2022-11-18

**Authors:** Bianca Maria Maglia Orlandi, Omar Asdrúbal Vilca Mejia, Jennifer Loría Sorio, Pedro de Barros e Silva, Marco Antonio Praça Oliveira, Marcelo Arruda Nakazone, Marcos Gradim Tiveron, Valquíria Pelliser Campagnucci, Luiz Augusto Ferreira Lisboa, Jorge Zubelli, Sharon-Lise Normand, Fabio Biscegli Jatene

**Affiliations:** 1 Department of Cardiovascular Surgery, Instituto do Coração do Hospital das Clínicas da Faculdade de Medicina da Universidade de São Paulo (INCOR), São Paulo, São Paulo Brazil; 2 Department of Health Care Policy, Harvard Medical School, Boston, USA; 3 Department of Cardiovascular Surgery, Hospital Samaritano Paulista, São Paulo, São Paulo Brazil; 4 Universidad de Costa Rica, San José, Costa Rica; 5 Instituto de Matemática Pura e Aplicada (IMPA), Rio de Janeiro, Brazil; 6 Department of Cardiovascular Surgery, Beneficência Portuguesa de São Paulo, São Paulo, São Paulo Brazil; 7 Faculdade de Medicina de São José do Rio Preto, São José de Rio Preto, São Paulo Brazil; 8 Department of Cardiovascular Surgery, Irmandade da Santa Casa de Misericórdia de Marília, Marília, São Paulo Brazil; 9 Department of Cardiovascular Surgery, Irmandade da Santa Casa de Misericórdia de São Paulo, São Paulo, São Paulo Brazil; 10 Khalifa University, Abu Dhabi, United Arab Emirates; 11 Department of Biostatistics, Harvard TH Chan School of Public Health, Boston, USA

Correction to: *Scientific Reports* 10.1038/s41598-022-19473-1, published online 07 September 2022

The original version of this Article contained an error in Affiliation 1, which was incorrectly given as:

Department of Cardiovascular Surgery, Instituto do Coração do Hospital das Clínicas da Faculdade de Medicina do Estado de São Paulo (INCOR), São Paulo, São Paulo, Brazil.

The correct Affiliation is listed below:

Department of Cardiovascular Surgery, Instituto do Coração do Hospital das Clínicas da Faculdade de Medicina da Universidade de São Paulo (INCOR), São Paulo, São Paulo, Brazil.

The original Article and the error in the Supplementary Information 1 file that accompanies the original Article have been corrected.

