# Supplementary material for: Performance of a novel risk model for deep sternal wound infection after coronary artery bypass grafting
Source: Sci Rep. 2022 Sep 7;12:15177. doi: 10.1038/s41598-022-19473-1 (PMC9452524; doi:10.1038/s41598-022-19473-1)
Supplement: Supplementary file 1 — Supplementary Tables. [file 41598_2022_19473_MOESM1_ESM.docx]

**Performance of a novel risk model for deep sternal wound infection after Coronary Artery Bypass Grafting**

**Bianca Maria Maglia Orlandi^1,2^; Omar Asdrubal Vilca Mejia^1,3,^*; Jennifer Loría Sorio^4,5^; Pedro de Barros e Silva^3,^+; Marco Antonio Praça de Oliveira^6,^+; Marcelo Arruda Nakazone^7,^+; Marcos Gradim Tiveron^8,^+; Valquíria Pelliser Campagnucci^9,^+; Luiz Augusto Ferreira Lisboa^1^; Jorge Zubelli^10^; Sharon-Lise Normand^2,11^; Fabio Biscegli Jatene^1^.**

^1^Department of Cardiovascular Surgery, Instituto do Coração do Hospital das Clínicas da Faculdade de Medicina da Universidade de São Paulo (INCOR), São Paulo, São Paulo, Brazil.

^2^Department of Health Care Policy, Harvard Medical School, Boston, United States.

^3^Department of Cardiovascular Surgery, Hospital Samaritano Paulista, São Paulo, São Paulo, Brazil.

^4^Universidad de Costa Rica, Costa Rica, America Central.

^5^Instituto de Matemática Pura e Aplicada (IMPA), Rio de Janeiro, Brazil.

^6^Department of Cardiovascular Surgery, Beneficência Portuguesa de São Paulo, São Paulo, São Paulo, Brazil.

^7^Faculdade de Medicina de São José do Rio Preto, São José de Rio Preto, São Paulo, Brazil.

^8^Department of Cardiovascular Surgery, Irmandade da Santa Casa de Misericórdia de Marília, Marília, São Paulo, Brazil.

^9^Department of Cardiovascular Surgery, Irmandade da Santa Casa de Misericórdia de São Paulo, São Paulo, São Paulo, Brazil.

^10^Khalifa University, Abu Dhabi, United Arab Emirates.

^11^Department of Biostatistics, Harvard TH Chan School of Public Health, Boston, United States.

+these authors contributed equally to this work.

*Corresponding author: email: [omar.mejia@incor.usp.br](mailto:omar.mejia@incor.usp.br).

Supplementary table 1. DEWINSCORE variables with statistical significance or clinical rationale included for LASSO regression model.

| 1.     Age (years) | 29.  Previous Percutaneous Coronary Intervention |
| --- | --- |
| 2.     Gender | 30.  Creatinine (mg/dL) |
| 3.     Body Mass Index ≥ 30kg/cm^2^ (categorical) | 31.  Creatinine clearance |
| 4.     Diabetes | 32.  INR (international normalized ratio) |
| 5.     Insulin dependent | 33.  Angina Class (Canadian Cardiovascular Society - CCS) |
| 6.     Renal Failure | 34.  Arrhythmia |
| 7.     Family History Coronary Artery Disease | 35.  Calcio blockers |
| 8.     Dyslipidemia | 36.  Anticoagulant therapy |
| 9.     Hypertension | 37.  Coronary artery disease |
| 10.  Bronchodilator therapy | 38.  Number of disease vessels |
| 11.  Pneumonia | 39.  Aortic Insufficiency |
| 12.  Depression | 40.  Mitral Insufficiency |
| 13.  Liver Disease | 41.  Tricuspid Insufficiency |
| 14.  Immunosuppress | 42.  Surgery Status (elective versus urgent) |
| 15.  Cancer | 43.  Pre-operative hospital time (days) |
| 16.  Thoracic Aortic Disease | 44.  Lowest Intra operative temperature (Celsius) |
| 17.  Syncope | 45.  Surgery duration (hours) |
| 18.  Chronic Lung Disease | 46.  Cardiopulmonary bypass time (minutes) |
| 19.  Peripheral arterial disease | 47.  Radial artery used |
| 20.  Cardiovascular disease | 48.  Intra-Aortic balloon pump |
| 21.  Hemoglobin (mg/dL) | 49.  Lowest Intra operative hemoglobin (mg/dL) |
| 22.  Hematocrit (%) | 50.  Highest Intra operative Glucose (mg/dL) |
| 23.  Leucocytes (/mm^3^) | 51.  Intra operative blood transfusion |
| 24.  Platelets (/mm^3^) | 52.  Bilateral Internal thoracic artery used |
| 25.  Previous Myocardial Infarction | 53.  Ejection Fraction (%) |
| 26.  Prior Heart failure | 54.  Antibiotic Prophylaxis (binary variable) |
| 27.  Cardiogenic Shock | 55.  Additional Antibiotic intraoperative |
| 28.  Resuscitation |  |

Supplementary table 2. Baseline characteristics among patients undergoing isolated CABG. REPLICCAR, São Paulo, Brazil, 2017-19.

| Variables | Missing |  | |
| --- | --- | --- | --- |
|  | % | n | % |
| Age (years)* | 0.0 | 63.26 ± 9.19 | |
| Male gender | 0.0 | 3,034 | 74.27 |
| BMI (kg/m^2^)* | 1.1 | 27.53 ± 4.33 | |
| Family history of CAD | 0.0 | 659 | 16.13 |
| Diabetes | 0.0 | 2,010 | 49.20 |
| Dyslipidemia | 0.0 | 2,515 | 61.57 |
| Renal failure | 0.0 | 296 | 7.25 |
| Dialysis | 0.0 | 81 | 1.98 |
| Hypertension | 0.0 | 3,602 | 88.18 |
| Rheumatic disease | 0.0 | 52 | 1.27 |
| Chagas disease | 0.0 | 19 | 0.47 |
| Chronic lung disease | 0.4 | 197 | 4.82 |
| Bronchodilator therapy | 0.0 | 68 | 1.66 |
| Peripheral vascular disease | 0.0 | 287 | 7.03 |
| Preoperative stroke | 4.4 | 163 | 3.99 |
| Previous MI | 0.2 | 2,162 | 52.93 |
| < 21 days | 14.8 | 738 | 18.1 |
| ≥ 21 days | 14.8 | 1,422 | 34.8 |
| Hemoglobin (mg/dL)* | 4.9 | 13.5 ± 1.8 | |
| Creatinine (mg/dL)* | 4.8 | 1.24 ± 1.1 | |
| Percutaneous coronary intervention | 4.0 | 624 | 15.3 |
| Angina CCS 4 | 5.9 | 423 | 10.5 |
| Heart failure | 5.4 | 388 | 9.5 |
| NYHA ≥ III | 4.9 | 573 | 14.0 |
| Cardiogenic shock | 0.3 | 23 | 0.6 |
| Inotropes | 0.0 | 29 | 0.7 |
| Insulin-dependent | 0.0 | 573 | 14.0 |
| Three-vessel disease | 14.2 | 1,075 | 26.3 |
| Ejection fraction (%)* | 19.8 | 57.6 ± 12.3 | |
| Elective surgery | 0.1 | 2,654 | 64.9 |
| Lowest intraoperative temperature (ºC)* | 10.3 | 33.9 ± 1.92 | |
| Surgery duration (hours)* | 1.2 | 4.77 ± 1.60 | |
| BITA | 0.0 | 469 | 11.5 |
| CPB time (minutes)* | 9.3 | 75.8 ± 29.3 | |
| IABP | 0.0 | 175 | 4.28 |

^*^ Mean ± SD; BMI: body mass index; CAD: coronary artery disease; MI: myocardial infarction; CCS: Canadian Cardiovascular Society; NYHA: New York Heart Association; BITA: bilateral internal thoracic artery; CPB: Cardiopulmonary bypass; IABP: intra-aortic balloon pump.
